# Downregulation of Macrophage-Specific Act-1 Intensifies Periodontitis and Alveolar Bone Loss Possibly via TNF/NF-κB Signaling

**DOI:** 10.3389/fcell.2021.628139

**Published:** 2021-03-04

**Authors:** Janak L. Pathak, Ying Fang, Yunxin Chen, Zhitong Ye, Xueqi Guo, Yongyong Yan, Jun Zha, Dongliang Liang, Xiuxian Ke, Luxi Yang, Wenchao Zhong, Lijing Wang, Liping Wang

**Affiliations:** ^1^Guangzhou Key Laboratory of Basic and Applied Research of Oral Regenerative Medicine, Affiliated Stomatology Hospital of Guangzhou Medical University, Guangzhou, China; ^2^Vascular Biology Research Institute, School of Life Sciences and Biopharmaceutics, Guangdong Pharmaceutical University, Guangzhou, China

**Keywords:** periodontitis, NF-κB activator 1, macrophages, inflammation, alveolar bone loss

## Abstract

Periodontitis is a chronic inflammatory oral disease that affects almost half of the adult population. NF-κB activator 1 (Act1) is mainly expressed in immune cells, including macrophages, and modulates immune cells’ function to regulate inflammation in inflammatory diseases. Macrophages play a vital role in the pathophysiology of periodontitis. However, the effect of macrophage-specific Act1 on periodontitis has not been investigated yet. This study aims to unravel the role of macrophage-specific Act1 on the pathophysiology of periodontitis. The expression of Act1 in healthy and periodontitis periodontal tissue was confirmed by immunohistochemistry. Macrophage-specific Act1 expression downregulated (anti-Act1) mice were developed by inserting anti-Act1 antisense oligonucleotides after the CD68 promoter of C57BL/6 mice. Ligature-induced periodontitis (LIP) was induced in anti-Act1 mice and wildtype mice. Micro-CT, histology, and TRAP staining analyzed the periodontal tissue status, alveolar bone loss, and osteoclast numbers. Immunohistochemistry, RT-qPCR, and ELISA analyzed the inflammatory cells infiltration, expression of inflammatory cytokines, and M1/M2 macrophage polarization. mRNA sequencing of *in vitro* bacterial lipopolysaccharide (LPS)-treated peritoneal macrophages analyzed the differentially expressed genes in anti-Act1 mice during inflammation. Anti-Act1 mice showed aggravated periodontitis and alveolar bone loss compared to wildtype. Periodontitis-affected periodontal tissue (PAPT) of anti-Act1 mice showed a higher degree of macrophage infiltration, and M1 macrophage polarization compared to wildtype. Levels of pro-inflammatory cytokines (IL-1β, IL-6, and TNFα), and macrophage activity-related factors (CCL2, CCL3, and CCL4) were robustly high in PAPT of anti-Act1 mice compared to wildtype. mRNA sequencing and KEGG analysis showed activated TNF/NF-κB signaling in LPS-treated macrophages from anti-Act1 mice. *In vitro* studies on LPS-treated peritoneal macrophages from anti-act1 mice showed a higher degree of cell migration and expression of inflammatory cytokines, macrophage activity-related factors, M1 macrophage-related factors, and TNF/NF-κB signaling related P-p65 protein. In conclusion, downregulation of macrophage-specific Act1 aggravated periodontitis, alveolar bone loss, macrophage infiltration, inflammation, and M1 macrophage polarization. Furthermore, LPS-treated macrophages from anti-Act1 mice activated TNF/NF-κB signaling. These results indicate the distinct role of macrophage-specific Act1 on the pathophysiology of periodontitis possibly via TNF/NF-κB signaling.

## Introduction

Periodontitis is a bacterially induced chronic inflammatory disease of the periodontium (Darveau, 2010). Periodontitis affects more than half of the adult population in China, the United States, and Europe (Holtfreter et al., 2010; Hu et al., 2011; Eke et al., 2012; Zhang et al., 2014). Disrupted interaction between the host immune defense mechanism and the oral microorganisms are frequently observed in periodontitis (Hajishengallis, 2014). In periodontitis, pro-inflammatory cytokines produced by immune cells amplify inflammation cascades in the surrounding tissues that eventually cause tissue destruction and tooth loss (Darveau, 2010; Vos et al., 2012). Currently available therapeutic interventions for periodontitis only alleviate the disease intensity and progression rather than the complete cure of the disease (Graziani et al., 2017). Therefore, novel cost-effective therapeutic approaches to treat periodontitis are in high demand. Due to the complex nature of the disease, the molecular mechanisms involved in periodontitis pathophysiology are still not clear. Therefore, an in-depth study of the molecular mechanisms and signaling pathways involved in periodontitis is essential to establish novel therapeutic targets for cost-effective treatments.

Various cell signaling molecules play role in immune cell-mediated inflammation modulation. Li et al. (2000) reported the role of NF-κB activator 1 (Act1) in inflammation modulation in 2000. Act1 is an intracellular protein with a molecular weight of 60 kD containing 574 amino acids and mainly expressed by macrophages, B cells, and T Cells (Qian et al., 2002; Novatchkova et al., 2003). A notable feature of Act1 is that it contains a TRAF6 binding sequence. Act1 mediates the ubiquitination of TRAF6 through its U-box region, and then activates the TAK1 kinase and IKK kinase complex, thereby activating the transcription factor NF-κB (Liu et al., 2009). Act1 regulates inflammation by modulating immune cell functions in a disease-specific manner. In autoimmune encephalomyelitis and allergic lung inflammation mouse models, the Act1 knockout inhibits IL-17-mediated inflammation (Swaidani et al., 2009). Act1 knockout (KO) mice show high numbers of peripheral B cells eventually leading to lymphadenopathy, splenomegaly, hyperglobulinemia, and increased autoantibodies (Qian et al., 2008). The role of macrophage-specific Act1 in inflammatory disease periodontitis is still unknown.

In the early period of periodontitis, macrophages play an important role in the immune response of periodontitis by controlling the pathogenicity of periodontal biofilm and activating adaptive immunity (Sima et al., 2019). In addition, macrophages and osteoclasts have a similar origin, and under inflammatory conditions, macrophages can transform to M1 phenotype and osteoclasts (Kubatzky et al., 2018; Pereira et al., 2018). M1 type macrophages produce pro-inflammatory cytokines and aggravate periodontitis (Yang et al., 2018). While M2 macrophages produce anti-inflammatory cytokines to alleviate periodontitis (Veloso et al., 2020). Clinical and animal studies had reported the role of a higher periodontal M1/M2 macrophages ratio in the pathophysiology of periodontitis (Yang et al., 2018; Zhu et al., 2019; Veloso et al., 2020). However, the role of Act1 on M1 and M2 macrophage polarization during inflammatory conditions such as periodontitis has not been investigated yet.

In this study, we aimed to investigate the role of macrophage-specific Act1 on periodontitis pathophysiology. We developed ligature-induced periodontitis (LIP) in macrophage-specific Act1 expression downregulated (anti-Act1) mice and extensively analyzed the disease status, inflammation, and alveolar bone loss. We analyzed the mRNA sequencing in bacterial lipopolysaccharide (LPS)-treated peritoneal macrophages from anti-Act1 mice to investigate the differential expression of migration and inflammation-related genes. Cell migration and proinflammatory cytokines release in LPS-treated peritoneal macrophages from anti-Act1 mice were further analyzed *in vitro*. Our results revealed that the downregulation of macrophage-specific Act1 aggravates periodontitis, alveolar bone loss, inflammation, M1-polarization, and TNF/NF-κB signaling.

## Materials and Methods

### Human Gingival Tissues Collection

Human gingiva, including sulcus/pocket epithelium and underlying connective tissue, were obtained from healthy controls (*n* = 6) during the wisdom tooth extraction. Patients with periodontitis with alveolar bone loss were confirmed by radiography and clinical observation. Gingival tissues from periodontitis patients (*n* = 6) were obtained during the treatment procedure of periodontitis. Patients and healthy controls’ clinical characteristics and demographics are summarized in Table 1. Written informed consent was obtained from each participant. The Medical Ethics Committee of the Affiliated Stomatology Hospital of Guangzhou Medical University approved all protocols dealing with patients.

**TABLE 1:** Clinical characteristics and periodontal status of healthy controls and periodontitis patients.

Gender/age	Simplified oral hygiene index (OHI-S)	Bleeding index (BI)	Tooth mobility (TM)	Probing depth (PD)/mm	Gingival recession (GR)/mm	Attachment loss (AL)/mm	Furcation index (FI)	Degree of periodontitis
Female/65	2	0–1	0	2	0	0	0	−
Male/65	2	0–1	0	2	0	0	0	−
Male/44	1	0	0	1–2	0	0	0	−
Female/43	1	0	0	2	0	0	0	−
Female/42	1–2	0–1	0	2	0	0	0	−
Male/40	1	0	0	2	0	0	0	−
Female/67	4	4	III°	6–8	4	4–6	II–III°	+++
Male/67	4	4	III°	6–8	4	4–6	II–III°	+++
Female/64	3	3–4	III°	7–8	3–4	5–7	III°	+++
Male/51	4	4	III°	8	4	6	III°	+++
Female/45	3	3	III°	7–8	3	5–6	II–III°	+++
Male/27	3	2–3	III°	6–7	2–3	4–5	II–III°	+++

*^+ + +^specifies severe periodontitis.*

### Development of Anti-Act1 Mice

Wildtype mice were purchased from Guangdong Medical Animal Experiment Center (Guangdong, China). Anti-Act1 mice were obtained from Prof. Lijing Wang’s lab, Vascular Biology Research Institute, School of Life Sciences and Biopharmaceutics, Guangdong Pharmaceutical University, Guangzhou 510006, China. Anti-Act1 mice were developed by inserting anti-Act1 antisense oligonucleotides after the CD68 promoter of C57BL/6 mice (Wang et al., 2019). CD68 promoter-based insertion of antisense oligonucleotides confirms mononuclear macrophage-specific antisense function. Antisense was used to cover the region from 100 bp before the ATG start region of the Act1 gene to 200 bp after ATG to establish C57BL/6 background antisense-Act1 (anti-Act1) gene-modified mice, which ultimately inhibits the transcription of the Act1 gene. The development of macrophage-specific anti-Act1 mice is illustrated in Supplementary Figure 1. Insertion of antisense was confirmed by PCR using forward primer: 5′-CTGGTGCAGACAGCCTAGCTG-3′; reverse primer: 5′-CCTGCGAGCTAAAGT CCTGGA-3′. The PCR product was separated in agarose gel electrophoresis and visualized using a gel imager (Gel Doc^TM^ XR+, BIO-RAD). The knockdown of macrophage-specific Act1 gene expression was confirmed by RT-qPCR in peritoneal macrophages. Primers used for RT-qPCR of Act1 are listed in Table 2.

**TABLE 2:** Primer used for RT-pPCR.

Gene	Primer sequence (5′–3′)
*Act1*	Forward: TCCCGTGGAGGTTGATGAATC
	Reverse: TCAGGGTGCCTTCTAAAGAAACT
*Il-6*	Forward: CTGCAAGAGACTTCCATCCAG
	Reverse: AGTGGTATAGACAGGTCTGTTGG
*Il-1*β	Forward: GAAATGCCACCTTTTGACAGTG
	Reverse: TGGATGCTCTCATCAGGACAG
*Tnf-*α	Forward: TGTCTCAGCCTCTTCTCATT
	Reverse: TGATCTGAGTGTGAGGGTCT
*Ccl2*	Forward: TTAAAAACCTGGATCGGAACCAA
	Reverse: GCATTAGCTTCAGATTTACGGGT
*Ccl3*	Forward: TTCTCTGTACCATGACACTCTGC
	Reverse: CGTGGAATCTTCCGGCTGTAG
*Ccl4*	Forward: TTCCTGCTGTTTCTCTTACACCT
	Reverse: CTGTCTGCCTCTTTTGGTCAG
*Gapdh*	Forward: GTGAAGGTCGGTGTGAACGG
	Reverse: TCCTGGAAGATGGTGATGGG

### Development of Ligature-Induced Periodontitis in Mice

A total of 50 wildtype C57/B16 and 50 anti-Act1 mice (8 weeks old, male, ∼25 g body weight) were used in this study. The Experimental Animal Ethics Committee of Guangzhou Medical University approved all animal care and study protocols (GY2020-001). Ligation-induced periodontitis was developed as described previously (Abe and Hajishengallis, 2013). Briefly, the mice were anesthetized by Isoflurane (RWD, Shenzhen, China) and fixed prone on the animal-specific fixed plate. The oral cavity was disinfected with 75% ethanol. A 5-0 silk thread (Johnson & Johnson, United States) was ligated around the left upper second molar and fixed with a surgical knot. All the knots were placed on the palatal side. After the ligation was completed, the mouse’s tongue was slightly pulled out of the mouth and placed in a warmer place until the mouse wakes up. The mice were fed with normal chow and free drinking water. The silk thread fixation was examined every day after the ligation. If the silk threads were loose or the thread knots were incomplete, mice were excluded from the experiment. The day of ligation was considered as day 0. After 7 days of ligation, mice were sacrificed by cervical dislocation. Periodontal tissue samples were collected and analyzed for changes in the pathophysiology of periodontitis.

### Histology

For histology and immunohistochemistry, the left upper jaw, including teeth, periodontal tissues, and alveolar bone, was separated and the blood was washed out with PBS. The specimens were immediately fixed in 4% paraformaldehyde at room temperature for 16 h and immersed in 10% EDTA (pH 7.4) for 24 days for decalcification. Decalcified specimens were dehydrated and embedded in paraffin. Tissue sections of 3 μm thickness were obtained using a rotary microtome (Thermo Scientific^TM^ HM 325, Thermo Fisher Scientific, Waltham, MA, United States). Tissue sections were stained with hematoxylin and eosin (H&E) staining. Images were captured using a light microscope (DM4000B-LED/DFC450, Leica, Wetzlar, Germany). The distance from the enamel-cementum boundary to the alveolar crest (CEJ-ABC) between the maxillary first and second molars was measured as a net bone loss on tissue sections, using Image J software (Wang et al., 2015).

### Immunohistochemistry

Heat-induced antigen retrieval was performed in the tissue section as described previously (Xiao et al., 2017). Methanol containing 3% H_2_O_2_ was added on top of the tissue section for 20 min to block the endogenous peroxidase. Tissue sections were then blocked with 10% bovine serum albumin (BSA) and incubated with primary antibodies: anti-Act1 (bs6202R, BIOSS, China), anti-CD34 (ab81289, Abcam, Cambridge, United Kingdom), anti-CD45 (20103-1-AP, Proteintech, United States), anti-F4/80(70076, CST, United States) or isotope controls for overnight at 4°C. After 3 times washing with PBS, tissue sections were incubated with a corresponding anti-rabbit/anti-mouse secondary antibody for 30 min at 37°C. Mayer’s hematoxylin was used as a counterstaining. Finally, all stained sections were dehydrated through a series of graded alcohol baths of increasing concentration, cleared in xylene, and mounted with coverslips. Stained tissue sections were visualized under a microscope (DM4000B-LED/DFC450, Leica, Wetzlar, Germany), and images were captured. The visual fields between the first molar and the second molar were photographed. The number of immunostaining-positive cells was counted in the entire area of each image.

### TRAP Staining

To visualize the osteoclasts, tissue sections were stained using a tartrate acid phosphatase (TRAP) kit (G1050, Servicebio, Wuhan, China). TRAP-stained histological tissue sections were examined under a microscope, and images of predefined areas were captured. TRAP-positive osteoclasts in the alveolar bone tissue section between the first molar and the second molar were counted.

### Micro CT Analysis

The left maxillary specimens fixed with paraformaldehyde were scanned by SkyScan1172 (Bruker-Micro-CT, Kontich, Belgium). The voxel resolution of the scanned volumes was 10 μm. Three-dimensional reconstruction was performed by SkyScan CTvox software after scanning. The SkyScan Dataviewer software was used to measure the buccal and palatal bone resorption heights of the maxillary second molars (Abe and Hajishengallis, 2013). The CEJ-ABC was regarded as the bone resorption height. The area of the same size at the bifurcation of the maxillary second molar was measured by SkyScan Dataviewer and CTAn software (version 1.17.7.2) for bone mineral density (BMD) analysis.

### Flow Cytometry Analysis

The cell suspension was prepared from mice periodontal tissue, including gingiva, periodontal ligament, and a part of alveolar bone as described previously (Wang L. et al., 2020). The cell suspension was used for flow cytometry analysis (Mizraji et al., 2013). Prior to the test, cells were counted and cell viability was evaluated by the Zombie NIR^TM^ Fixable Viability Kit (423105,Biolegend, Beijing, China). Cells were stained with 1 μg of PE anti-mouse F4/80 (123110, BioLegend, San Diego, CA, United States) solution, FITC anti-mouse/human CD11b (101206, BioLegend, San Diego, CA, United States), PerCP/Cyanine5.5 anti-mouse CD16/32 (101324, BioLegend, San Diego, CA, United States), APC anti-mouse CD206 (141707, BioLegend, San Diego, CA, United States) or anti-rabbit IgG per 1 × 10^6^ cells for 30 min on ice in the dark. After two times washing with PBS, the cells were resuspended in 300 μl PBS and transferred to flow tubes. Flow cytometry analysis was performed using the FACSAria III Cell Sorter (BD Biosciences, San Jose, CA, United States). The mononuclear cells were sorted using anti-CD11b. From mononuclear cells, macrophages were sorted using anti-F4/80. And from macrophages, M1 and M2 macrophages were sorted using CD16/32 and CD 206, respectively.

### Real-Time qPCR Analysis

The left maxillary specimen was kept in a dry cryotube, immersed in liquid nitrogen, and stored at −80°C. Frozen tissue was poured into a mortar pre-cooled with liquid nitrogen-containing 1 ml of lysis solution added with 1% β-thioethanol and quickly ground into the lysate. Total RNA was isolated from the lysate using the PureLink^TM^ RNA Mini kit (Cat no.12183018A, Thermo Fisher Scientific, United States). RNA concentration and quality were measured in the NanoDrop spectrophotometer (NanoDrop2000, Wilmington, DE, United States). Total RNA was reverse-transcribed using Takara PrimeScript^TM^ RT Master Mix in T100 Thermal Cycler (Bio-Rad, United States). Expressions of specific genes were analyzed by real-time qPCR (RT-qPCR) using Takara TB-Green Premix Ex Taq in the LightCycler RT-qPCR system (LC-480 II, Roche, Switzerland). Gapdh was used as a reference housekeeping gene. The 2^–ΔΔ^Ct method was used to analyze the relative mRNA expression levels. The primers used for RT-qPCR are listed in Table 2.

### mRNA Sequencing

Peritoneal macrophage from wildtype and anti-Act1 mice was isolated and cultured as described previously (Pineda-Torra et al., 2015). The macrophages were treated with 100 ng/ml bacterial LPS (tlrl-pglps LPS-PG, InvivoGen, United States) for 24 h to mimic *in vivo* inflammatory milieu. The cultures were used for RNA sequencing. Total RNA was extracted from peritoneal macrophage cell culture using the Trizol kit (Invitrogen, Carlsbad, CA, United States), according to the manufacturer’s protocol. The RNA concentration was determined using Qubit, and the RNA amount and purity of each sample was assessed with a NanoDrop spectrophotometer. RNA was isolated in 40 μl of DEPC water and stored at −80°C. Briefly, RNA-seq libraries were prepared by using the Illumina Truseq^TM^ RNA sample prep Kit and were sequenced using an Illumina HiSeq. Cutadapt was used to obtain paired-end reads (Kechin et al., 2017). The reads were aligned with Hisat2 (v 2.1.0) to GRCm38 with default parameters (Kim et al., 2015). Only the data matched to the reference genome were used for subsequent analysis. The mapped reads of each sample were assembled using StringTie (Pertea et al., 2015). Then, the transcriptome from all samples was merged to reconstruct a comprehensive transcriptome using Perl scripts. After the final transcriptome was generated, StringTie (Pertea et al., 2015) and Ballgown (Frazee et al., 2015) were used to estimate the expression levels of all transcripts. Traditional singular enrichment analysis (SEA) was used for the enrichment analysis of Kyoto Encyclopedia of Genes and Genomes (KEGG) pathways (Rodriguez et al., 2016). The enrichment *p*-value calculation was performed with Fisher’s exact test.

### Enzyme-Linked Immunosorbent Assay

Enzyme-linked immunosorbent assay (ELISA) was performed to measure the protein expression in periodontal tissue protein extract, conditioned medium of mice macrophages culture, and mice macrophages cell lysates. The macrophages were treated with 100 ng/ml LPS for 24 h. The periodontal tissue cell lysate was prepared as described previously (Wang L. et al., 2020). Mice peritoneal macrophages were isolated as described previously (Kheirandish et al., 2018). On the first day of macrophages culture, 100 ng/ml LPS was added. After 24 h of LPS treatment, conditioned medium (CM) was collected, and attached cells in the culture harvested for cell lysate preparation. The protein levels of IL-6, IL-1β, and TNF-a in periodontal tissue protein extract and CM were measured by mouse ELISA Standard Kit (RayBiotech, Atlanta, United States). The protein levels of CD16/32, CD206, and MCP-I in cell lysates were measured by Mouse ELISA Standard Kit (Cusabio, Wuhan, China).

### Western Blot Analysis

Cell lysates from peritoneal macrophages treated with 100 ng/ml LPS for 24 h were prepared using RIPA buffer containing protease PMSF and phosphatase inhibitor. The protein samples (20 μl) were separated by 10% SDS-PAGE and transferred onto polyvinylidene fluoride (PVDF) membranes (EMD Millipore, Billerica, MA, United States). PVDF membranes were blocked with 5% reconstituted dry milk at 37°C for 2 h and incubated with the primary antibody p-65 (1:10000 dilution, Cell Signaling Technology, Beverly, MA, United States), phosphorylated P-p65 (1:10000 dilution, Cell Signaling Technology) at 4°C overnight, followed by secondary antibodies (1:5000 dilution, Cell Signaling Technology) at room temperature for 1 h. Membranes were washed three times with TBS with 0.05% Tween-20. An enhanced chemiluminescence detection system BLT GelView 6000 Pro (BioLight, Guangdong, China) detected the immunoreactive protein bands. Protein expression levels were evaluated using β-actin as a loading control.

### Macrophage Migration Assay

Cell migration assay was performed using Transwell chambers (8.0 μm pore size; Corning, United States). The peritoneal macrophages were trypsinized and cell suspension (1 × 10^6^/ml) in DMEM medium with 10% FBS was prepared. Cell suspension (200 μl) was added in the upper chamber and add 700 μl of DMEM medium containing 10% FBS to the lower chamber to culture for 2 h. The upper chamber was replaced with the DMEM, 100 ng/ml LPS, and no FBS. The lower chamber was replaced with DMEM containing 10% FBS serum. After 24 h incubation, non-migrated cells in the upper chamber were removed with a cotton swab, and migrated cells were fixed in 4% paraformaldehyde and stained with 0.5% crystal violet. Photographs were taken randomly from five fields of each membrane. The number of invading cells was expressed as the average number of cells per microscopic field over five fields.

### Statistical Analysis

All data are presented as mean ± standard deviation (SD). Statistical analysis was performed using GraphPad Prism 7.0 statistical software (GraphPad, San Diego, CA, United States). For where appropriate (comparison of two groups only), two-tailed *t*-tests were done. *p* < 0.05 was considered to indicate a statistically significant difference.

## Results

### The Expression of Act1 in Periodontal Tissues and Macrophages

**FIGURE 1:**
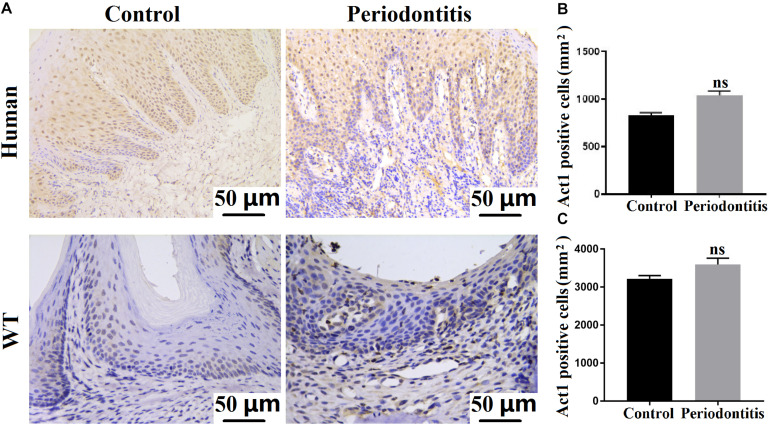
Act1 is expressed in healthy and periodontitis gingival tissue. **(A)** Representative immunohistochemistry images of healthy/periodontitis gingival tissue (human and mice) show Act1 expression. Quantitative analysis of Act1 positive cells in healthy and periodontitis human gingival tissue **(B)**, and mice periodontal tissue **(C)**. Data are presented as mean ± SD (*n* = 6). WT, wildtype.

We examined the expression of Act1 in human gingival tissues from patients with periodontitis and age and sex-matched healthy individuals. Both periodontitis and healthy gingiva showed a similar extent of Act1 expression (Figures 1A,B). We further tested the expression pattern of Act1 in periodontal tissue of healthy and periodontitis mice. The result of Act1 expression in periodontal tissue of healthy and periodontitis mice was per the results from human gingiva (Figures 1A–C). Meticulous examination of histological images indicated that the Act1 is mainly expressed in the epithelium of healthy human gingiva and mice periodontitis-affected periodontal tissue (PAPT) (Figure 1A). However, Act1 expression was observed in both the epithelium and connective tissue of human gingiva and mice PAPT during periodontitis (Figure 1A).

We analyzed the macrophage-specific Act1 expression in human gingival tissue during periodontitis. Act1 was expressed in macrophages infiltrated in gingiva during periodontitis (Supplementary Figure 2A). Immunofluorescence staining of mice peritoneal macrophages showed Act1 expression (Supplementary Figure 2B). Immunohistochemistry of mice PAPT also showed robust macrophage-infiltration (Supplementary Figure 2C).

### Knockdown of Macrophage-Specific Act1 Aggravates Periodontitis

Act1 had been reported to play a vital role in various inflammatory diseases (Pisitkun et al., 2010; Swaidani et al., 2011; Doyle et al., 2012; Wang et al., 2013; Sakamuri et al., 2016). Furthermore, our results indicated Act1 expression in macrophages infiltrated in gingiva and PAPT during periodontitis. Therefore, we further analyzed the role of macrophage-specific Act1 in the pathophysiology of periodontitis. The expression of Act-1 in macrophages of anti-Act1 mice was downregulated by twofold (Supplementary Figure 1B). We induced periodontitis in wildtype and anti-Act1 mice and extensively analyzed the role of macrophage-specific Act1 in the pathophysiology of periodontitis.

**FIGURE 2:**
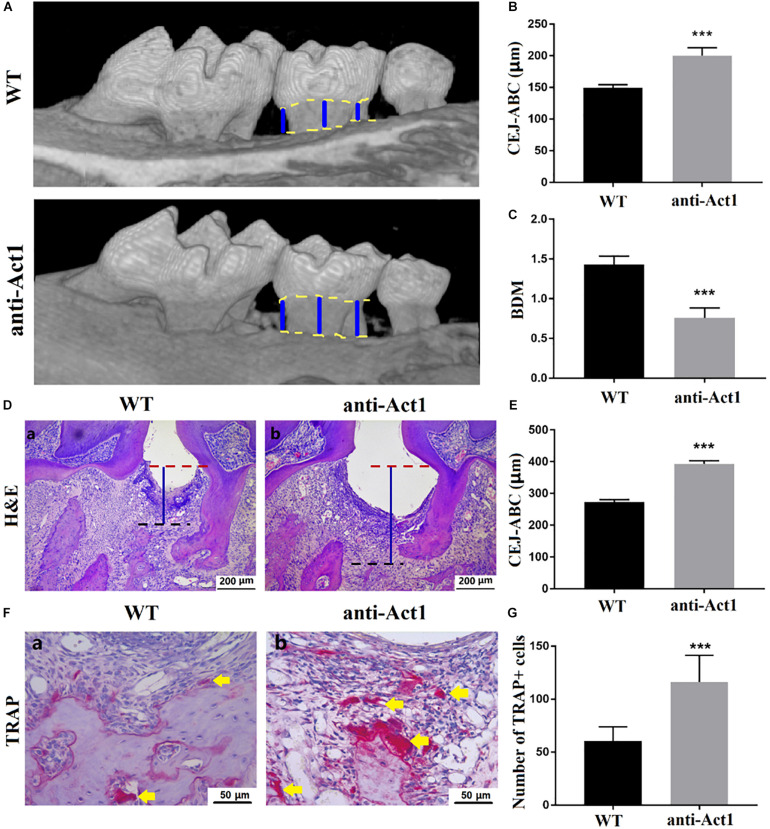
Periodontitis and alveolar bone loss were aggravated in anti-Act1 mice compared to wildtype. **(A)** Micro-CT images of the healthy and periodontitis-affected maxilla of wildtype and anti-Act1 mice. Quantitative analysis of CEJ-ABC distance **(B)**, BMD **(C)** from micro-CT images. **(D)** H&E stained histological images of PAPT of wild-type and anti-Act1 mice. **(E)** Quantitative analysis of CEJ-ABC distance from histological images. **(F)** TRAP stained histological images of PAPT of wild-type and anti-Act1 mice. **(G)** Quantitative analysis of osteoclasts in PAPT. Data are presented as mean ± SD (*n* = 6). The significant difference between the groups, ***P < 0.001. CEJ, cement-enamel junction; ABC, alveolar bone crest; Red dot line, CEJ level; Black dot line, ABC level; blue line, CEJ-ABC distance; yellow arrow, osteoclasts; WT, wild-type; and BMD, bone mineral density.

Micro-CT images of periodontitis maxilla showed a higher degree of periodontal tissue and alveolar bone loss in anti-Act1 mice compared to wildtype mice (Figure 2A). CEJ-ABC distance increases with the degree of periodontitis. The CEJ-ABC distance in anti-Act1 periodontitis mice was 1.3-fold higher compared to wildtype periodontitis mice (Figure 2B). Alveolar bone mineral density was decreased by twofold in anti-Act1 periodontitis mice compared to wildtype periodontitis mice (Figure 2C). H&E stained histological images of PAPT showed a higher degree of periodontal tissue and alveolar bone loss in anti-Act1 periodontitis mice compared to wildtype periodontitis mice (Figure 2D). The gingival epithelium was intact in PAPT of wildtype mice. But, the gingival epithelium was degraded in PAPT anti-Act1 mice (Figure 2D). The result of periodontal tissue destruction and alveolar bone loss from histological analysis supports the findings from the micro-CT analysis. Moreover, the CEJ-ABC distance analysis from histological images was per the result analyzed from micro-CT images (Figure 2E). Higher osteoclast activity is responsible for alveolar bone loss during periodontitis. TRAP staining in PAPT histological tissue section revealed robustly higher numbers of osteoclasts in the anti-Act1 periodontitis-group (Figure 2F). Quantitative analysis of osteoclasts in the histological tissue section showed twofold higher numbers of osteoclasts in the anti-Act1 periodontitis mice compared to wildtype periodontitis mice (Figure 2G).

### Knockdown of Macrophage-Specific Act1 Stimulates Macrophage Infiltration in PAPT

**FIGURE 3:**
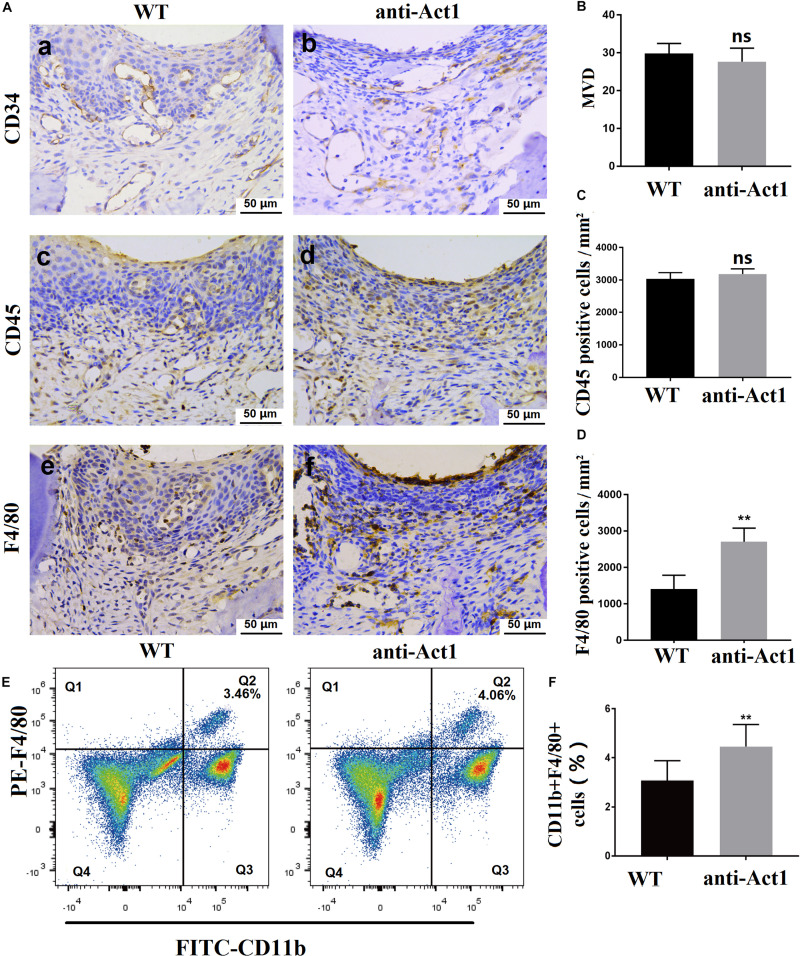
Macrophage infiltration in PAPT was enhanced in anti-Act1 mice compared to wildtype. **(A)** Immunohistochemistry images of PAPT. Quantitative analysis of CD34 expressing MVD **(B)**, CD45 expressing pan leucocytes **(C)**, and F4/80 expressing macrophages **(D)**, *n* = 6. **(E)** FACs images of macrophages sorting using FITC-CD11b and PE-F4/80. **(F)** Quantitative FACs data. Calculated the ratio of CD11b + F4/80+ cells, *n* = 8. The significant difference between the groups, **P < 0.01, ns, not significant. MVD, Microvessel density; WT, wildtype.

Angiogenesis in periodontal tissue is increased during periodontitis. CD34 expressing newly formed blood vessels were observed in PAPT of wildtype and anti-Act1 periodontitis mice (Figure 3A). However, there was no difference in microvessel density between these two groups (Figure 3B). Leukocyte infiltration in periodontal tissue is frequently observed in periodontitis. A similar degree of leukocyte infiltration was observed in PAPT of wildtype and anti-Act1 mice (Figures 3A,C). Macrophages are the key immune cells involved in the pathophysiology of periodontitis. The number of macrophages infiltrated in PAPT was robustly high (twofold) in anti-Act1 mice compared to wildtype (Figures 3A,D). FACs analysis of mononuclear cells from PAPT also showed higher numbers (1.2-fold) of macrophages in PAPT of anti-Act1 mice compared to wildtype (Figures 3E,F).

### Anti-Act1 Mice Show Higher Expression of Proinflammatory Cytokines and Macrophage Migration-Specific Factors in PAPT

**FIGURE 4:**
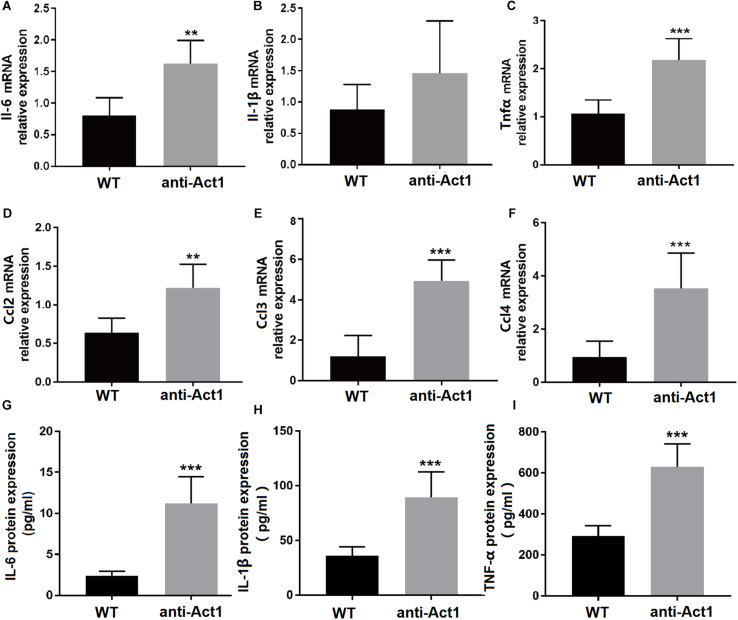
**(A–F)** mRNA level expression of inflammatory cytokines and macrophage migration-related factors in PAPT of wildtype and anti-Act1 mice. **(G–I)** Protein level expression of inflammatory cytokines in PAPT of wildtype and anti-Act1 mice. Data are presented as mean ± SD (*n* = 6). The significant difference between the groups, **P < 0.01, ***P < 0.001. WT, wildtype.

RT-qPCR of total RNA from PAPT tissue showed 2.3-fold higher expression of Il-6 in the anti-Act1 group compared to wildtype (Figure 4A). There was no significant difference on the mRNA expression of Il-1β between these groups (Figure 4B). Tnfα expression was 2.0-fold higher in PAPT of anti-Act1 mice compared to wildtype (Figure 4C). Ccl2, Ccl3, and Ccl4 are the macrophage migration markers. PAPT of anti-Act1 mice showed robustly higher mRNA expression of macrophage migration markers compared to wildtype (Figures 4D–F). Monocyte chemotactic protein 1 (MCP1 or CCL2), macrophage inflammatory protein 1 (MIP-1α or CCL3), and MIP-1β (CCL4) are crucial for macrophage migration/invasion and immune responses toward infection and inflammation. The expression of Ccl2, Ccl3, and Ccl4 in PAPT of anti-Act1 mice was, respectively, 2. 0-, 5. 0-, and 3.2-fold higher compared to wild type (Figures 4D–F). This result indicates the inflammation modulatory activity of macrophage-specific Act1 in periodontitis.

### LPS-Treated Peritoneal Macrophages From Anti-Act1 Mice Show the Differential mRNAs Expression Pattern

**FIGURE 5:**
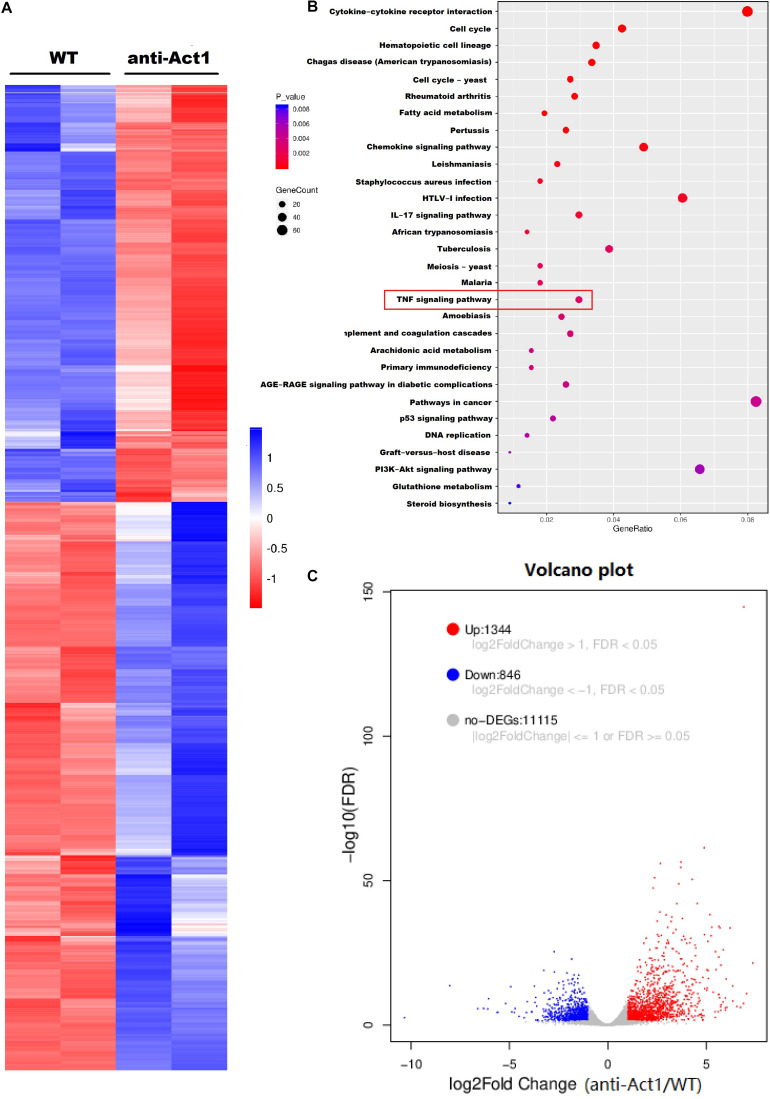
Lipopolysaccharide -treated peritoneal macrophages from wildtype and anti-Act1 mice showed a differential expression of 2190 mRNAs. **(A)** Heat map, **(B)** KEGG pathway analysis, and **(C)** Volcano map showing differentially expressed mRNAs. Red color intensity indicates upregulation, and blue color intensity indicates the downregulation of mRNA expression.

mRNA sequencing was performed to analyze the effect of Act1 knockdown on differential expression of mRNAs in macrophages during inflammation. Peritoneal macrophages from anti-Act1 and wildtype mice were treated with LPS to mimic *in vivo* inflammatory conditions. A total of 1,344 mRNAs were differentially upregulated and 846 were differentially downregulated in LPS-treated macrophages from Act-1 knockdown mice compared to macrophages from wildtype mice (Figure 5A). Figure 5B indicates the KEGG pathway analysis from differentially expressed mRNAs identifying the affected signaling pathways. TNF-signaling plays a vital role in inflammation modulation in periodontitis via NF-κB signaling (Van Quickelberghe et al., 2018; Yang et al., 2020). The TNF-signaling pathway was among the top 30 pathways detected in KEGG analysis indicating its’ role in macrophage-specific Act1-mediated modulation of inflammation. Figure 5C illustrates the Volcano plot of non-affected as well as differentially expressed genes between LPS-treated macrophages from anti-Act1 and wildtype mice.

### LPS-Treated Macrophages From Anti-Act1 Mice Express a Higher Level of Inflammatory Cytokines

**FIGURE 6:**
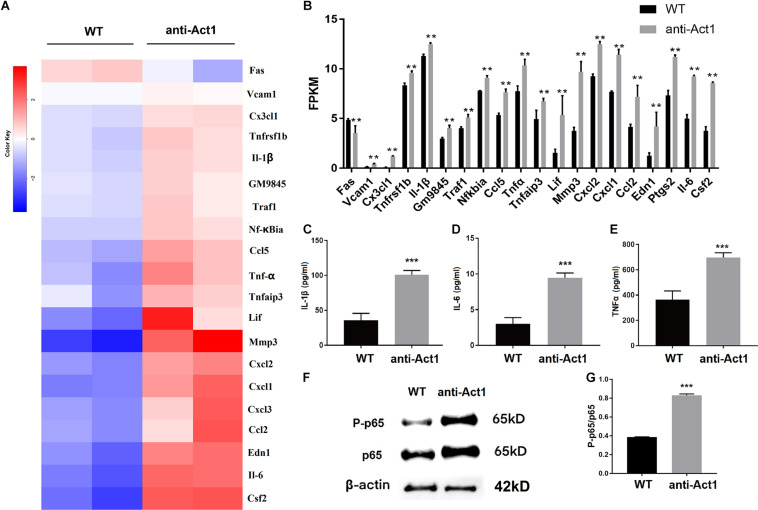
Lipopolysaccharide -treated peritoneal macrophages from anti-Act1 mice differentially upregulate the mRNA expression of TNF-NF-κB signaling pathway-related proteins. **(A)** Heat map, and **(B)** bar figures showing differentially upregulated mRNAs. **(C–E)** Protein level expressions of inflammatory cytokines in LPS-treated peritoneal macrophages from wildtype and anti-Act1 mice (measured by ELISA), *n* = 6. **(F)** Representative western bolt images of p65 and P-p65 expression. **(G)** Quantification of western blots (*n* = 3). Data are presented as mean ± SD (*n* = 6). The significant difference between the groups, **P < 0.01, ***P < 0.001. WT, wildtype. Red color intensity indicates upregulation, and blue color intensity indicates the downregulation of gene expression.

Based on the result from KEGG analysis, the differential gene expression of TNF-signaling-related proteins was further analyzed (Figure 6). Most of the TNF- signaling-related genes including, *Tnf*α, *Il6, Il-1*β, *and Ccl2* were differentially upregulated in LPS-treated macrophages from anti-Act1 mice compared to wildtype (Figures 6A,B). ELISA in conditioned medium from macrophage culture with LPS showed 2. 5-, 3. 0-, and 2.0-fold higher expression of IL-1β, IL-6, and TNFα in the anti-Act1 group compared to wildtype (Figures 6C–E). Western blot analysis showed robustly high expression of NF-κB related p65 and P-p65 protein in LPS-treated macrophages from anti-Act1 mice compared to wildtype (Figures 6F,G). These findings support the results from the RT-qPCR and ELISA analysis in PAPT from anti-Act1 and wildtype mice (Figure 4). These results indicate increased M1 macrophage polarization in the Act1-knockdowned group during the inflammation.

### LPS-Treatment Enhances Migration and M1-Polarization of Macrophages From Anti-Act1 Mice

**FIGURE 7:**
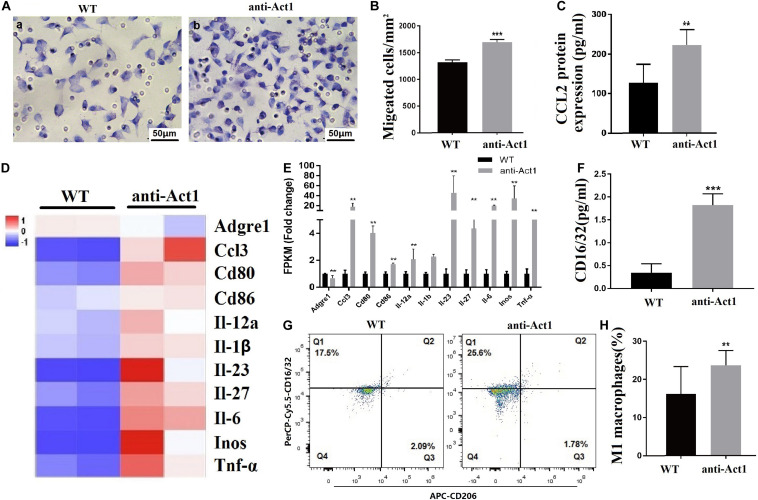
Lipopolysaccharide -treated peritoneal macrophages from anti-Act1 mice showed higher migration and M1-polarization. **(A)** Representative images of macrophage migration. **(B)** Quantitative analysis of macrophage migration, *n* = 3. **(C)** CCL2 protein expression (ELISA, *n* = 6). Heat map **(D)**, and bar figures of differentially expressed macrophage M1-polarization-related genes **(E)**. **(F)** CD16/32 protein level expression analyzed by ELISA, *n* = 6. **(G)** FACs images of mice periodontal macrophages sorting using CD16/32 specific antibody. **(H)** Quantitative FACs data, *n* = 6. The significant difference between the groups, **P < 0.01, ***P < 0.001. WT, wildtype. Red color intensity indicates upregulation, and blue color intensity indicates the downregulation of gene expression.

Macrophage infiltration in gingiva and periodontal tissue plays a vital role in the pathophysiology of periodontitis. LPS-treated anti-Act1 peritoneal macrophages showed a higher rate of migration compared to wildtype macrophages (Figures 7A,B). Macrophage migration-related MCP-1 protein (CCL2) expression was also enhanced by 1.8-fold in LPS-treated anti-Act1 peritoneal macrophages compared to wildtype (Figure 7C). This result was in accordance with the *Mcp-1* mRNA expression in PAPT of anti-Act1 mice (Figure 4D) and mRNA sequencing data from LPS-treated macrophages from anti-Act1 mice (Figures 6A,B).

M1 macrophages are inflammatory in nature and produce proinflammatory cytokines, including, IL-1β, IL-6, and TNFα (Yuan et al., 2017; Yang et al., 2018; Hasegawa et al., 2019). Since expression levels of inflammatory cytokines were robustly upregulated in PAPT of anti-Act1 mice and LPS-treated anti-Act1 peritoneal macrophages, we further examined the M1-macrophage polarization. mRNA sequencing data showed differentially upregulated M1-macrophage related genes (Figures 7D,E). ELISA in cell lysate showed 5.0-fold higher expression of M1 macrophage marker CD16/32 in LPS-treated anti-Act1 peritoneal macrophages compared to that of wildtype (Figure 7F). FACs analysis of F4/80 positive macrophages from PAPT showed a higher number of M1 macrophages in anti-Act1 mice compared to wildtype mice (Figures 7G,H). In contrast, M2 macrophage-related genes were downregulated or remained unchanged in LPS-treated anti-Act1 peritoneal macrophages (Supplementary Figures 3A,B). Moreover, the protein expression of M2 macrophage marker CD206 in PAPT of anti-Act1 mice was reduced compared to wildtype (Supplementary Figure 3C).

## Discussion

**FIGURE 8:**
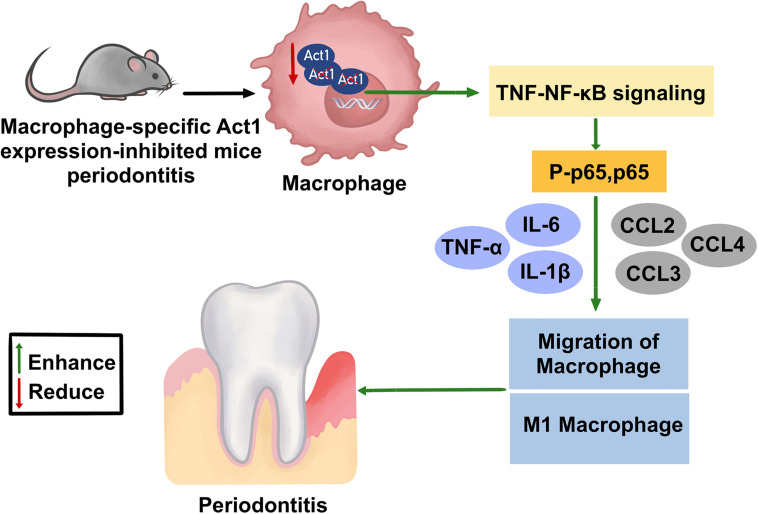
Effect of macrophage-specific downregulation of Act1 on the pathophysiology of mice periodontitis.

The global prevalence rate of severe periodontitis is ∼10% (Kassebaum et al., 2014). The macrophages are a key immune cell that regulates the pathophysiology of periodontitis (Sima et al., 2019). Act1 is an intracellular signaling molecule that modulates inflammation in various diseases (Pisitkun et al., 2010; Swaidani et al., 2011; Doyle et al., 2012; Sakamuri et al., 2016). However, the role of macrophage-specific Act1 on the pathophysiology of periodontitis has not been investigated yet. In this study, we developed macrophage-specific Act1 expression inhibited mice and analyzed its role in the pathophysiology of mice periodontitis. These Anti-Act1 mice showed severe periodontitis and alveolar bone loss compared to wildtype. Macrophage infiltration, levels of inflammatory cytokines such as IL-6, IL-1β, and TNFα, and M1 macrophage polarization were enhanced in anti-Act1 periodontitis mice compared to wildtype periodontitis mice. Furthermore, TNF/NF-κB signaling was induced in macrophages from anti-Act1 mice under inflammatory conditions. These results confirmed the involvement of macrophage-specific Act1 on the pathophysiology of periodontitis possibly via regulating inflammation and M1 macrophage polarization (Figure 8).

Act1 was expressed in both healthy and periodontitis oral tissue in humans and mice. Act1 is expressed in various tissues, including the brain, heart, kidney, skeletal muscle, and bone marrow (Li et al., 2000; Xia et al., 2002). Alveolar bone resorption, periodontal tissue degeneration, higher CEJ-ABC, severe alveolar bone loss, and an increased number of osteoclasts were observed in anti-Act1 periodontitis mice compare to wildtype periodontitis mice. A higher degree of angiogenesis and infiltration of leucocyte/macrophages in PAPT have been reported in severe cases of periodontitis (Shi et al., 2008; Parisi et al., 2018; Wang L. et al., 2020; Zhang Z. et al., 2020). In this study, a similar extent of neo-vasculogenesis and leucocyte infiltration was observed in anti-Act1 and wildtype LIP mice. However, macrophage infiltration was highly enhanced in PAPT of anti-Act1 mice compared to wildtype. M1 macrophage polarization is associated with the severity of periodontitis (He et al., 2020; Wang L. et al., 2020). In this study, M1 macrophage polarization was enhanced in PAPT of anti-Act1 mice compared to wildtype. M1 macrophages release the pro-inflammatory cytokines amplifying inflammation in the PAPT milieu (He et al., 2020; Wang L. et al., 2020). The higher levels of pro-inflammatory cytokines in PAPT aggravate periodontitis and alveolar bone loss (Fredriksson et al., 2002; Azuma et al., 2014; Sakalauskiene et al., 2016; Wang D. et al., 2020). Chemotactic molecules CCL2, CCL3, and CCL4 play roles in the infiltration of macrophages and other immune cells in PAPT (Repeke et al., 2010; Parisi et al., 2018). In this study, levels of inflammatory cytokines IL-6, IL-1β, TNFα, and chemotactic molecules CCL2, CCL3, and CCL4 were robustly higher in PAPT of anti-Act1 mice than wildtype. Furthermore, levels of inflammatory cytokines, such as IL-6, IL-1β, and TNFα, and the expression of M1 macrophage polarization related genes were enhanced in macrophages from anti-Act1 mice under the inflammatory condition *in vitro*. These results further confirm the role of macrophage-specific Act-1 on periodontitis via immune modulation, inflammation regulation, and immune cell migration.

NF-κB regulates periodontal inflammation and the pathophysiology of periodontitis (Francis et al., 2020). TNF/NF-κB signaling also plays a role in the pathophysiology of various inflammatory diseases (Liedtke and Trautwein, 2012; Kunz, 2013; Sayegh et al., 2019). mRNA sequencing of LPS-treaded macrophages from anti-Act1 mice showed upregulation of TNF/NF-κB signaling related genes, including Tnfα, Il-1β, Ccl2, and Nfkbia. KEGG analysis revealed the activation of TNF/NF-κB signaling in LPS-treated macrophages from anti-Act1 mice. Shreds of literature had reported that the activated TNF/NF-κB signaling enhances M1-macrophage polarization (Liu et al., 2017; Lu et al., 2020; Zhang H. et al., 2020). Inflammation triggers M1 macrophage polarization and M1 macrophages further release proinflammatory cytokines thereby amplifying inflammation in a vicious fashion in the periodontitis milieu. This M1 macrophage-induced inflammation could be responsible for the abrogated periodontitis in anti-Act1 mice. Elevated levels of proinflammatory cytokines in periodontal tissue affect the osteoblast activity that might partly contribute to the alveolar bone loss (Lacey et al., 2009). The reports from the literature and results of this study indicate the role of macrophage-specific Act1 on the pathophysiology of periodontitis possibly via TNF/NF-κB signaling-mediated M1 macrophage polarization.

Act1 regulates the function of various other cells, including epithelial cells, B cells, and T cells. Overexpression of Act1 leads to the activation of NF-κB and JNK signaling in epithelial cells (Qian et al., 2002). Act1 is a negative regulator in T cell and B cell (Zhang et al., 2018). Shreds of literature had reported the role of Act1 in various inflammatory diseases (Qian et al., 2007, 2008; Li, 2008; Swaidani et al., 2009; Kang et al., 2010; Zhang et al., 2018). Compared with IL-17-stimulated wild-type mice, Act1-deficient mice have reduced recruitment of neutrophils to the airways and Act1 deficiency in epithelial cells reduces the phenotype of allergic lung inflammation (Swaidani et al., 2009). The deficiency of Act1 leads to the development of Sjögren’s syndrome (Qian et al., 2008). In mouse models of experimental autoimmune encephalomyelitis (EAE) and asthma, the lack of Act1 leads to resistance to IL-17-mediated inflammation (Qian et al., 2007; Kang et al., 2010). Although Act1 is necessary for IL-17-mediated inflammation, Act1-deficient mice develop spontaneous SLE and Sjögren-like diseases (Li, 2008; Zhang et al., 2018). However, the role of Act1 in periodontitis and periodontitis-related inflammation has not been reported so far. This is the first study to report the role of macrophage-specific Act1 on the pathophysiology of periodontitis. Aggravated inflammation, alveolar bone loss, macrophage infiltration, and M1-macrophage polarization in anti-Act1 periodontitis mice also indicate the possible role of macrophage-specific Act-1 on bone and cartilage degenerative inflammatory diseases such as osteoarthritis, rheumatoid arthritis, and osteoporosis.

In this study, we developed LIP in macrophage-specific Act1 expression inhibited mice to investigate the role of macrophage-specific Act1 on the pathophysiology of periodontitis. Periodontal degeneration, alveolar bone loss, levels of inflammatory cytokines, immune cell infiltration, and M1 macrophage polarization in PAPT were extensively studied. *In vitro* studies related to macrophage infiltration, M1 macrophage polarization, mRNA sequencing, and inflammation were performed in macrophages from anti-Act1 mice to further validate the results from *in vivo* studies. Our results show that the macrophage-specific Act1 knockdown aggravates periodontitis possibly via activation of TNF/NF-κB signaling. The results of this study suggest the macrophage-specific Act1 as a possible target to treat periodontitis. However, future studies focusing on the pathophysiology of periodontitis in macrophage-specific Act1 overexpressed mice are necessary to prove this hypothesis. A limitation of this study is the lack of molecular mechanism of TNF/NF-κB activation in macrophages from anti-Act1 mice under inflammatory conditions. Currently, we are developing macrophage-specific Act1 transgenic mice to further investigate the role of macrophage-specific Act1 on the pathophysiology of periodontitis and underlying molecular mechanisms.

## Conclusion

Act1 is expressed in both healthy and periodontitis human gingival tissue as well as healthy and periodontitis periodontal tissue of mice. Downregulation of macrophage-specific Act1 in mice aggravated periodontitis, alveolar bone loss, and macrophage infiltration and M1 polarization. Anti-Act1 periodontitis mice showed a higher number of osteoclasts, and elevated level of pro-inflammatory cytokines and macrophage activity-related factors. Moreover, the LPS-treated macrophages from anti-Act1 mice activated TNF/NF-κB signaling and P-p65 expression. These results indicate the distinct role of macrophage-specific Act1 on the pathophysiology of periodontitis. Our findings warrant further studies to unravel the molecular mechanisms involved in macrophage-specific Act1-mediated regulation of periodontitis.

## Data Availability Statement

RNAseq data was uploaded to NCBI SRA database (SRA accession: PRJNA702259), https://www.ncbi.nlm.nih.gov/bioproject/PRJNA702259/.

## Ethics Statement

The studies involving human participants were reviewed and approved by the Medical Ethics Committee of the Affiliated Stomatology Hospital of Guangzhou Medical University. The patients/participants provided their written informed consent to participate in this study. The animal study was reviewed and approved by the Experimental Animal Ethics Committee of Guangzhou Medical University.

## Author Contributions

LJW, LPW, YF, and JP: study design. YC, ZY, XG, and YY: experimental conduct. JZ, YC, DL, XK, and LY: animal work. JZ, JP, YC, DL, LY, and WZ: data collection, analysis, and interpretation. YC and JP: manuscript preparation. All authors approved the final version of the manuscript.

## Conflict of Interest

The authors declare that the research was conducted in the absence of any commercial or financial relationships that could be construed as a potential conflict of interest.

## Supplementary Material

Supplementary Figure 1 **(A)** Scheme of macrophage-specific downregulation of Act1 in anti-Act1 mice. **(B)** Image PCR-product gel run showing a successful insertion of Act1 antisense oligonucleotides in anti-Act1 mice. **(C)** RT-qPCR data showing reduced Act-1 expression in macrophages from anti-Act1 mice. Data are presented as mean ± SD (*n* = 6). The significant difference between the groups, ^∗∗∗^*P* < 0.001. WT, wildtype.

Supplementary Figure 2 The expression pattern of Act1 in periodontal tissues and macrophages. Representative image of immunofluorescence staining showing the Act1 expression in macrophages in healthy human gingival tissue sections **(A)**, and peritoneal macrophages of wildtype mice **(B)**. **(C)** Macrophage infiltration in PAPT of wildtype mice. Act1-Alexa 488: green fluorescence, F4/80-Alexa 594: red fluorescence, CD68-Alexa 594: red fluorescence, DAPE: blue fluorescence, white arrow: Act1 is expressed in macrophages.

Supplementary Figure 3 **(A)** Heat map showing differential expression of M2 macrophage related genes. **(B)** Quantification of the expression level of M2 macrophage related genes from mRNA-seq data. **(C)** CD206 protein level expression in PAPT analyzed by ELISA, *n* = 6. Data are presented as mean ± SD. The significant difference between the groups, ^∗^*P* < 0.05. WT, wildtype.
