# Supplementary figures and images for: Downregulation of Macrophage-Specific Act-1 Intensifies Periodontitis and Alveolar Bone Loss Possibly via TNF/NF-κB Signaling
Source: Front Cell Dev Biol. 2021 Mar 4;9:628139. doi: 10.3389/fcell.2021.628139 (PMC7969798; doi:10.3389/fcell.2021.628139)

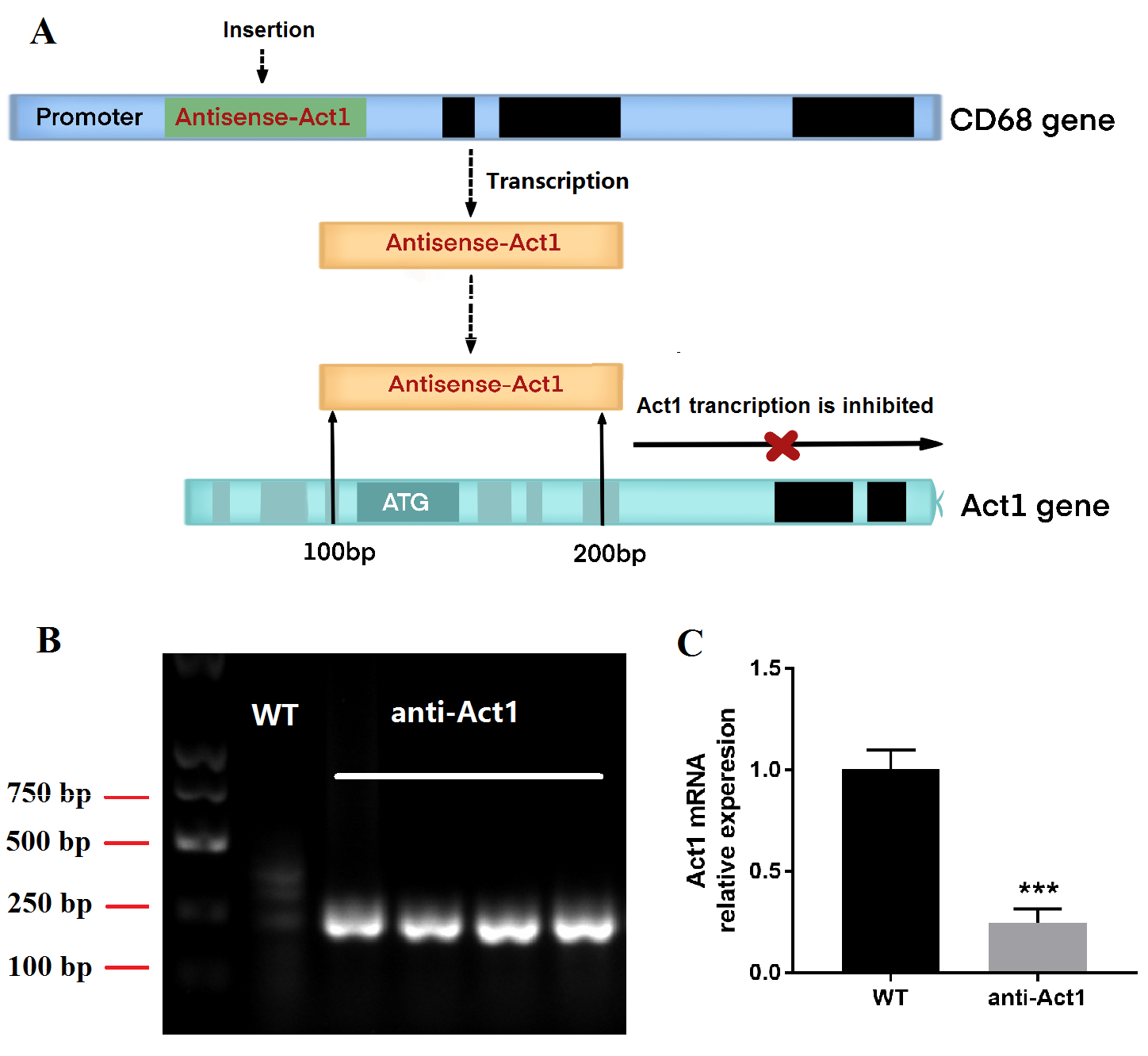

Supplement: Supplementary Figure 1 — (A) Scheme of macrophage-specific downregulation of Act1 in anti-Act1 mice. (B) Image PCR-product gel run showing a successful insertion of Act1 antisense oligonucleotides in anti-Act1 mice. (C) RT-qPCR data showing reduced Act-1 expression in macrophages from anti-Act1 mice. Data are presented as mean ± SD (n = 6). The significant difference between the groups, ∗∗∗P < 0.001. WT, wildtype. [file Image_1.TIF]

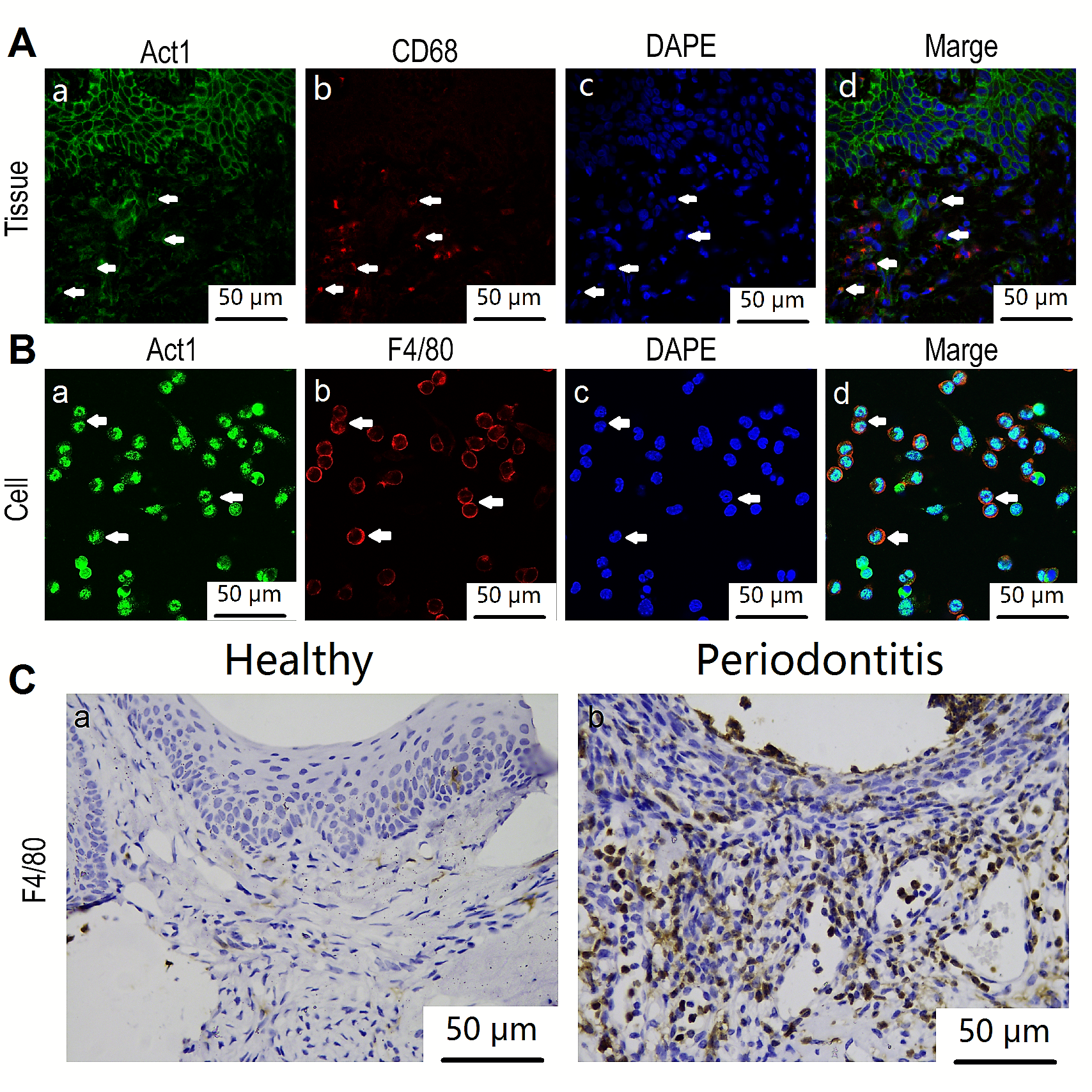

Supplement: Supplementary Figure 2 — The expression pattern of Act1 in periodontal tissues and macrophages. Representative image of immunofluorescence staining showing the Act1 expression in macrophages in healthy human gingival tissue sections (A), and peritoneal macrophages of wildtype mice (B). (C) Macrophage infiltration in PAPT of wildtype mice. Act1-Alexa 488: green fluorescence, F4/80-Alexa 594: red fluorescence, CD68-Alexa 594: red fluorescence, DAPE: blue fluorescence, white arrow: Act1 is expressed in macrophages. [file Image_2.TIF]

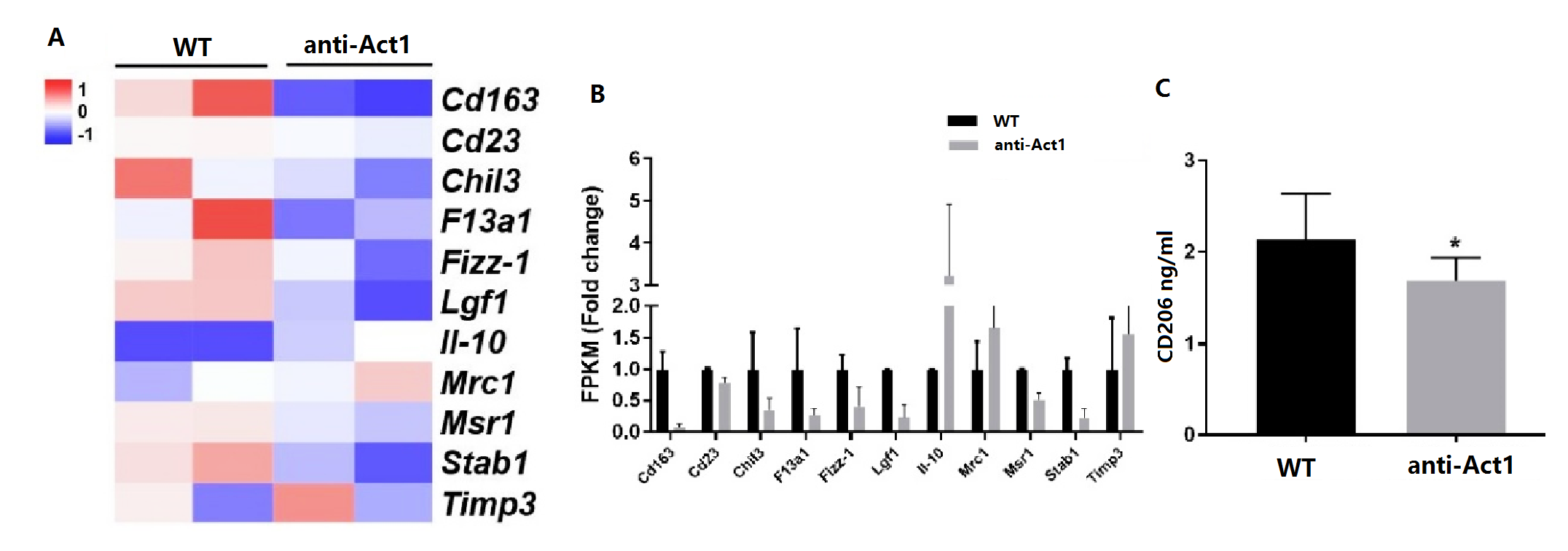

Supplement: Supplementary Figure 3 — (A) Heat map showing differential expression of M2 macrophage related genes. (B) Quantification of the expression level of M2 macrophage related genes from mRNA-seq data. (C) CD206 protein level expression in PAPT analyzed by ELISA, n = 6. Data are presented as mean ± SD. The significant difference between the groups, ∗P < 0.05. WT, wildtype. [file Image_3.TIF]
